# Cuproptosis and Diabetic Osteoporosis: Mechanisms and Therapeutic Prospects

**DOI:** 10.3390/ijms27031307

**Published:** 2026-01-28

**Authors:** Shih-Yu Chen, Wanyi Chen, Ze Gao, Yan Cui, Xiaofeng Zhu

**Affiliations:** 1 The First Affiliated Hospital of Jinan University, Guangzhou 510630, China; ensyuu_29@163.com (S.-Y.C.); 18235273687@163.com (Z.G.); 2 School of Traditional Chinese Medicine, Jinan University, Guangzhou 510630, China; 3 School of the First Clinical Medical, Jinan University, Guangzhou 510630, China; enwany@163.com; 4 The Affiliated Guangdong Second Provincial General Hospital of Jinan University, Guangzhou 510317, China

**Keywords:** cuproptosis, copper metabolism, copper homeostasis, diabetic osteoporosis

## Abstract

Diabetic osteoporosis (DOP) is a metabolic bone disease characterized by abnormal bone tissue structure and reduced bone strength in patients with diabetes. Its pathogenesis is complex, involving multiple factors rather than a single cause, and has not yet been fully elucidated. Cuproptosis, a novel form of programmed cell death discovered in 2022, differs mechanistically from apoptosis, necroptosis, and ferroptosis. This process relies on the accumulation of intracellular copper ions and is closely associated with mitochondrial respiration. Studies have indicated that cuproptosis is intimately linked to glucose metabolism and bone metabolism. This review explores the role of copper homeostasis in maintaining glucose metabolism and bone quality and systematically elucidates the potential associations between cuproptosis and these processes from molecular, cellular, and pathophysiological perspectives, aiming to provide new insights and prospects for future research directions in diabetic osteoporosis.

## 1. Introduction

Diabetic osteoporosis (DOP) is a metabolic bone disease caused by diabetes, primarily manifested as disruption of bone microstructure and decreased bone strength [1], which significantly increases the risk of fractures [2,3]. Its core mechanisms involve hyperglycemia-induced accumulation of advanced glycation end products (AGEs), oxidative stress, chronic inflammation, insulin deficiency or resistance, and decreased IGF-1 levels, collectively leading to weakened bone formation and enhanced resorption. Additionally, abnormalities in calcium and phosphorus metabolism, vitamin D deficiency, and diabetic complications (such as nephropathy and neuropathy) further exacerbate bone loss.

Copper, as an essential trace element, participates in the regulation of oxidative stress and energy metabolism, serving as a cofactor for various enzymes [4,5]. Recently discovered, cuproptosis is a copper-dependent form of cell death that mechanistically differs from classical death forms such as apoptosis, pyroptosis, and necroptosis [6]. Its uniqueness lies in the direct action of copper ions on lipoylated proteins in the tricarboxylic acid (TCA) cycle, inducing abnormal oligomerization and proteotoxic stress [6]; simultaneously, it leads to the loss of iron–sulfur cluster proteins, thereby triggering mitochondrial metabolic crisis and cell death [7].

Current clinical interventions for DOP primarily focus on glucose-lowering and calcium supplementation, but long-term application has limitations and adverse effects [8]. Copper homeostasis not only influences glucose metabolism but also participates in bone quality maintenance, and its dysregulation may be closely associated with the onset and progression of DOP [9,10,11]. Targeting the cuproptosis process and regulating copper homeostasis hold promise as a novel therapeutic strategy for DOP, with high research value and translational potential. This narrative review searched the PubMed database (from January 1978 to September 2025) using the following search strategy: (cuproptosis OR “copper induced cell death”) AND (diabetes OR osteoporosis). Inclusion criteria comprised original studies, reviews, and mechanistic studies; exclusion criteria included non-English literature and conference abstracts. A total of 122 references were included.

## 2. Cuproptosis

### 2.1. Copper Metabolism and Uptake

**Figure 1:**
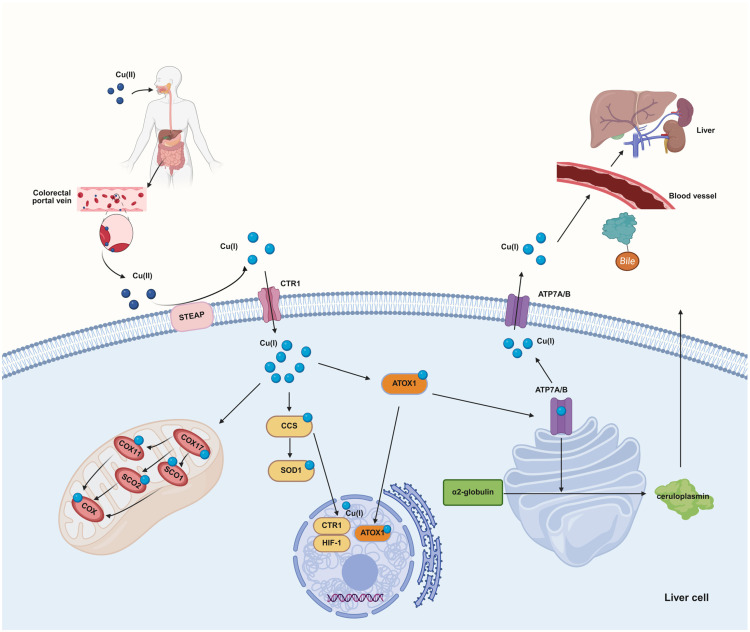
The metabolic pathway of copper. Created in BioRender. Chen, W. (2026) https://BioRender.com/ldp5o5g.

Copper is an essential trace element in the human body, primarily stored in muscles, bones, the liver, and blood. Copper is excreted from the body via the biliary system, with a small portion reabsorbed and utilized in the intestines, while the majority is eliminated in feces [7,12,13,14,15]. Dietary copper mainly exists as Cu^2+^; however, this form cannot be directly absorbed and utilized by cells. The surface of gastrointestinal epithelial cells contains various reductases that reduce Cu^2+^ to Cu^+^. After reduction, copper ions can bind to copper transporter 1 (CTR1) and are then absorbed by intestinal epithelial cells [16]. Following absorption, copper ions (Cu^+^) are released into the portal circulation via copper-transporting ATPase 1 (ATP7A). Most newly absorbed copper ions enter hepatocytes through the action of CTR1 protein [17]. In hepatocytes, copper chaperone antioxidant protein 1 (Atox1) and copper-transporting ATPase 2 (ATP7B) mediate the binding of the Golgi complex to α2-globulin (i.e., apoceruloplasmin), thereby forming ceruloplasmin, which is subsequently transported to various systems throughout the body [18,19]. The copper chaperone CCS is responsible for transporting Cu^+^ to SOD1 protein, which catalyzes the degradation of superoxide radicals and plays a key role in combating oxidative stress. The absorption, distribution, storage, and excretion of copper ions collectively regulate copper metabolic balance. Both excessively high and low copper ion levels can lead to various diseases. Disruption of copper balance results in intracellular copper ion accumulation, causing cellular damage and ultimately leading to cell death [20] (Figure 1).

### 2.2. Morphological Features and Biochemical Functions of Cuproptosis

The typical morphological features of cuproptosis include mitochondrial shrinkage, cell membrane rupture, endoplasmic reticulum damage, and chromatin fragmentation. These features resemble those of apoptosis, but the underlying mechanisms differ from other known forms of cell death. In copper-treated retinal cells, the endoplasmic reticulum membrane thins, forming large vacuoles, while the endoplasmic reticulum exhibits a loose structure [21]. Beyond morphological characteristics, the biochemical mechanisms underlying cuproptosis are complex and multifaceted. Under normal conditions, cells maintain copper ion concentrations at low levels through active homeostatic mechanisms. However, excessive copper ion levels lead to cell death. Therefore, intracellular copper ion levels are a key indicator for detecting cuproptosis. The tricarboxylic acid (TCA) cycle is involved in the cuproptosis process. Notably, various metabolites in the TCA cycle also aid in detecting cuproptosis, such as the accumulation of α-ketoglutarate and the reduction of succinate [6]. Key target proteins for detecting cuproptosis include FDX1, DLAT, DLST, HSP70, LIAS, Fe-S ligands, ATP7A/B, and SLC31A1 [22].

### 2.3. Key Conditions for Cuproptosis Initiation

**Figure 2:**
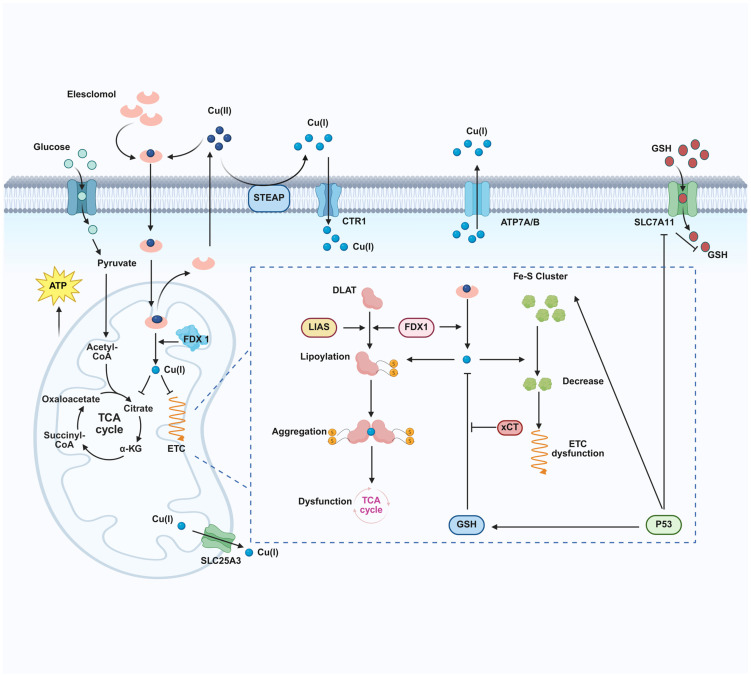
Copper-induced cell death mechanism. Created in BioRender. Chen, W. (2026) https://BioRender.com/2mf6mmd.

The mechanisms of general copper-induced oxidative stress and apoptosis are distinct from those of cuproptosis. The classical copper toxicity pathway begins with the excessive accumulation of intracellular free copper ions. Copper ions act as Fenton reaction catalysts, generating large amounts of reactive oxygen species (ROS), which attack and damage key biomacromolecules such as lipids, proteins, and DNA. When oxidative damage exceeds cellular repair capacity, it triggers increased mitochondrial outer membrane permeability, leading to cytochrome c release, which activates the caspase cascade and ultimately results in caspase-dependent apoptosis [23,24,25]. In contrast, cuproptosis is a novel mechanism independent of ROS or classical apoptosis executors. Its core feature lies in the direct interaction of copper ions with lipoylated tricarboxylic acid (TCA) cycle proteins, regulated by a highly specific genetic program. The reductase FDX1 has been identified as the most critical direct upstream regulator of cuproptosis. FDX1 not only reduces Cu^2+^ to the more toxic Cu^+^ but also promotes the lipoylation modification of key enzymes, ultimately leading to proteotoxic stress and mitochondrial metabolic dysfunction [6]. Under copper overload conditions, copper ions directly bind to these lipoylated TCA cycle proteins, inducing their oligomerization and functional loss [26,27]. This process disrupts the normal function of the mitochondrial respiratory chain, causing proteotoxic stress and ultimately rapid cell death. Notably, this pathway is independent of caspase activation and cannot be inhibited by classical apoptosis or necroptosis inhibitors [28] (Figure 2).

### 2.4. Comparison of Cuproptosis with Traditional Cell Death

#### 2.4.1. Cuproptosis vs. Ferroptosis

Ferroptosis, discovered in 2012, is an iron-dependent form of programmed cell death primarily driven by iron overload and lipid peroxide accumulation [29]. Ferroptosis is also closely related to metal metabolism and involves alterations in mitochondrial function, but there are significant differences in molecular mechanisms and morphological features between the two. Mechanistically, the core of ferroptosis is the inactivation of glutathione peroxidase 4 (GPX4), leading to the inability to promptly clear phospholipid hydroperoxides, thereby causing membrane lipid damage. This process relies on iron-mediated Fenton reactions, generating large amounts of reactive oxygen species (ROS) and resulting in oxidative stress. In contrast, although cuproptosis also involves abnormal accumulation of metal ions (copper), its primary mechanism is through the direct binding of copper ions to lipoylated proteins in the TCA cycle, leading to proteotoxic stress rather than oxidative stress. Morphologically, ferroptotic cells exhibit mitochondrial shrinkage, increased mitochondrial membrane density, and reduced or disappeared cristae, whereas cuproptotic cells mainly show mitochondrial contraction and mitochondrial membrane rupture. These two death forms also differ in key regulatory molecules: ferroptosis is regulated by factors such as GPX4, ACSL4, and FTH1, while cuproptosis is dominated by molecules like FDX1, DLAT, and LIAS.

#### 2.4.2. Cuproptosis vs. Apoptosis

Apoptosis is the earliest extensively studied form of programmed cell death, mediated by the caspase family and activated through death receptor pathways, mitochondrial pathways, or endoplasmic reticulum stress pathways [30]. Compared to cuproptosis, apoptosis exhibits significant differences in both morphological and biochemical characteristics. Apoptotic cells primarily show cell membrane shrinkage, chromatin condensation, and apoptotic body formation, ultimately being cleared by phagocytes without inducing an inflammatory response. In contrast, cuproptosis mainly involves mitochondrial metabolic dysfunction, does not form typical apoptotic bodies, and may be accompanied by a certain degree of inflammatory response, although its inflammation level is lower than that of pyroptosis. Molecularly, the core executors of apoptosis are the caspase protease family (e.g., Caspase-3, -8, and -9) and BCL-2 family proteins (e.g., Bax, Bcl-2). In contrast, the key regulatory molecules of cuproptosis are FDX1 and lipoylated proteins (e.g., DLAT, DLST). Additionally, apoptosis is typically triggered by signals such as DNA damage or growth factor deprivation, whereas cuproptosis is induced by specific metal ion (copper) overload, accompanied by a particular metabolic state (high mitochondrial respiration).

#### 2.4.3. Cuproptosis vs. Pyroptosis

Pyroptosis is an inflammatory form of programmed cell death, primarily executed by gasdermin family proteins and accompanied by the release of large amounts of pro-inflammatory factors [31]. Unlike cuproptosis, the core characteristic of pyroptosis is its intense inflammatory response, which is particularly important in infectious and inflammatory diseases. Mechanistically, pyroptosis activates Caspase-1 (classical pathway) or Caspase-4/5/11 (non-classical pathway) via inflammasomes, subsequently cleaving GSDMD protein to release its N-terminal domain, forming pores in the cell membrane, leading to cell osmotic lysis and the release of pro-inflammatory factors such as IL-1β and IL-18. In contrast, cuproptosis does not directly depend on Caspase activation or GSDMD cleavage but instead triggers cell death through metabolic disturbances within mitochondria.

#### 2.4.4. Differences Between Cuproptosis in DOP and Traditional Cell Death Mechanisms

In diabetic osteoporosis, traditional cell death mechanisms (such as apoptosis) and cuproptosis collectively contribute to impaired bone formation, albeit through distinct pathways. In traditional cell death, high glucose and insulin resistance primarily induce osteoblast apoptosis by triggering mitochondrial damage and endoplasmic reticulum stress. This process is dependent on the caspase signaling pathway, resulting in a net loss of osteoblasts. Copper-induced cell death presents a distinct mechanism of direct metabolic toxicity. The pathological environment of diabetes may disrupt copper homeostasis, leading to abnormal accumulation of copper ions in osteoblasts. The excess copper directly targets the lipoylated proteins in the mitochondrial tricarboxylic acid cycle, causing their aggregation and functional loss, thereby fundamentally impairing osteoblast function by disrupting cellular energy metabolism. Thus, a key distinction emerges: while apoptosis functions as “programmed clearance,” cuproptosis acts as “metabolic intoxication.” They synergistically exacerbate bone formation defects in diabetes from different dimensions (Table 1).

### 2.5. Association of Cuproptosis with Various Pathological Conditions

#### 2.5.1. Cancer

The relationship between copper and cancer is complex and of great interest. Studies have found that copper homeostasis dysregulation exists in various tumor tissues, and copper levels are closely associated with tumor proliferation, angiogenesis, and metastasis [32]. Cuproptosis plays a dual role in tumor biology [33]. On the one hand, tumor cells may utilize the cuproptosis mechanism to cope with metabolic stress during rapid growth; on the other hand, inducing cuproptosis has emerged as a promising anticancer strategy, particularly for tumors resistant to traditional chemotherapy.

#### 2.5.2. Neurodegenerative Diseases

Neurodegenerative diseases, such as Alzheimer’s disease, Parkinson’s disease, and Huntington’s disease, are chronic disorders characterized by progressive neuronal loss. Recent studies have found that copper homeostasis imbalance and cuproptosis mechanisms in the brain may play important roles in the pathogenesis of these diseases [34]. To address copper homeostasis imbalance in neurodegenerative diseases, researchers are exploring various copper-targeted therapeutic strategies. These include using copper chelators (e.g., penicillamine, tetrathiomolybdate) to remove excess copper and applying copper ionophores to induce cuproptosis in diseased cells under specific conditions [32]. Notably, due to the blood–brain barrier, the delivery of these drugs to the central nervous system faces challenges, and developing copper-regulating drugs that can efficiently cross the blood–brain barrier is a current research focus.

#### 2.5.3. Metabolic Diseases

Metabolic diseases refer to disorders caused by abnormal metabolic processes in the body, including but not limited to diabetes, obesity, and hypertension. The core of cuproptosis lies in mitochondrial metabolism, thus presenting potential links with metabolic disorders [35]. Copper homeostasis imbalance and abnormal activation of the cuproptosis pathway play significant roles in the pathogenesis of NAFLD, diabetes, cardiovascular diseases, and obesity by impairing the functions of hepatocytes, pancreatic β-cells, vascular endothelial cells, and adipocytes. Both inhibiting cuproptosis to protect key cells and activating it to eliminate pathological cells demonstrate substantial therapeutic potential.

## 3. Role of Cuproptosis in Glucose Metabolism and Osteoporosis

### 3.1. Copper and Glucose Metabolism

Copper bidirectionally regulates glucose homeostasis through dose-dependent mechanisms, with its action closely linked to cuproptosis. At physiological concentrations (0.5–5 μM), copper functions as a cofactor for key metabolic enzymes, whereas pathological accumulation (>10–20 μM) triggers FDX1-mediated cuproptosis by targeting lipoylated tricarboxylic acid (TCA) cycle proteins (DLAT/DLST) and disrupting mitochondrial iron–sulfur clusters [6,36]. This contrasts with copper-induced oxidative stress, which activates parallel death pathways (apoptosis, necroptosis) through the generation of reactive oxygen species (ROS) [37].

Dietary sugars influence copper absorption and toxicity. High-fructose diets suppress duodenal CTR1 expression, thereby reducing copper absorption and leading to mild copper deficiency, which exacerbates metabolic syndrome [38,39]. Conversely, in diabetic patients, hyperglycemia increases serum copper levels (>17.2 μM) and alters copper distribution: bound copper in plasma decreases, while free Cu^2+^ increases, promoting ROS formation and the accumulation of advanced glycation end products (AGEs) [27,40]. Clinical studies have shown a positive correlation between plasma copper levels, glycated hemoglobin (HbA1c) levels, and overall mortality in patients with type 1 and type 2 diabetes [41,42].

Copper ions bind to lipoylated TCA cycle enzymes, causing proteotoxic stress and impairing mitochondrial respiration [3]. In diabetic patients, the stabilization of HIF-1α exacerbates this situation, as HIF-1α shifts glucose metabolism toward glycolysis and aggravates copper-dependent mitochondrial dysfunction [43]. Notably, serum and urinary copper levels are associated with diabetic complications [44], indicating that copper imbalance is a biomarker and pathogenic factor in DOP.

### 3.2. Copper and Osteoporosis

Approximately two-thirds of the body’s copper is stored in muscles and bones. Copper plays a crucial role in regulating bone metabolism [44,45]. It is essential for maintaining mitochondrial respiratory chain function, scavenging free radicals, and promoting collagen cross-linking, which are vital for skeletal development, joint stability, and muscle contractility [46,47,48]. Additionally, copper directly inhibits osteoclast resorption [49,50]. The cellular effects of copper are dose-dependent: low doses promote osteoblast proliferation, whereas high doses may exert toxic effects on osteoblasts [51]. Notably, under copper deficiency (<10 μM serum copper), lysyl oxidase activity decreases, leading to impaired bone collagen cross-linking [52,53], while osteoclast activity remains relatively preserved [54,55]. At physiological copper levels (10–20 μM), mesenchymal stem cells (MSCs) are promoted to differentiate into osteoblasts, with upregulated Runx2 expression [56,57]. In mouse bone marrow macrophages induced to form osteoclasts, Cu^2+^ treatment for 72 h resulted in a 50% reduction in mature osteoclast numbers at 20 μM, as shown by TRAP staining [36]. Under copper overload (>25 μM), osteoclastogenesis is directly inhibited, but at >30 μM, it suppresses MSC osteogenic differentiation via the HIF-1α pathway [57,58].

Copper metabolic imbalance alters bone status by affecting osteoblast and osteoclast functions. In copper-deficient individuals, skeletal changes are primarily attributed to osteoblast dysfunction, while osteoclast activity appears unaffected. This leads to reduced osteoblast activity, impairing bone tissue remodeling processes, resulting in decreased bone mass, reduced bone strength, impaired bone formation and growth, reduced bone mineralization, and impaired ossification at growth centers [45,53]. Cuproptosis also alters the bone marrow microenvironment, impairing the osteogenic differentiation of bone marrow mesenchymal stem cells (BMSCs) and promoting adipogenic differentiation [59]. Adequate dietary copper intake is recommended to maintain bone density and reduce the risk of osteoporosis.

### 3.3. Pathophysiology of Copper in Diabetic Osteoporosis

Epidemiological studies have found that diabetic patients have a significantly higher risk of developing osteoporosis compared to non-diabetic individuals. This condition manifests as reduced bone density, degenerative bone structure, and increased fracture risk, thereby affecting patients’ quality of daily life [60,61,62,63]. As a severe skeletal complication of diabetes, the pathogenesis of diabetic osteoporosis involves multiple factors, including hyperglycemia, oxidative stress, accumulation of advanced glycation end products (AGEs), and chronic inflammation [64,65]. Recent studies indicate that copper homeostasis imbalance and its induction of cuproptosis play a key role in the pathogenesis of diabetic osteoporosis. In diabetic patients, disordered copper metabolism and disrupted skeletal homeostasis form a complex pathological network that accelerates bone loss.

In diabetic patients, copper levels are significantly elevated [66,67,68], which is closely associated with an increased risk of diabetes [69]. Copper stimulates the production of insulin-like growth factor (IGF) and growth hormone-releasing peptide (GHRP), thereby reducing blood glucose levels [40]. Excess copper generates reactive oxygen species, induces lipid peroxidation, disrupts bone metabolism, and exacerbates inflammatory responses [70,71,72,73,74,75,76]. Copper plays a positive role in promoting bone formation. However, in patients with diabetic osteoporosis, the opposite effect is observed, which may be related to their excessively high copper levels [45,77,78]. In terms of osteoporosis, copper exerts positive regulatory effects on bone metabolism-related cells and promotes the differentiation of mesenchymal stem cells into the osteoblastic lineage [45]. Existing studies have confirmed that within a certain dose range, increasing copper concentration enhances osteoblast activity and proliferation, whereas excessive copper concentrations cause toxic damage to osteoblasts [79]. These findings suggest an association between copper levels and the onset and progression of diabetic osteoporosis.

The hypoxic environment and glycolytic state in bone may play important roles in inhibiting cuproptosis [80,81]. In the bone microenvironment of diabetic patients, hypoxic areas often form due to reduced vascular distribution and inadequate blood supply. This hypoxic condition may promote a shift from mitochondrial respiration to glycolytic metabolism by inducing the HIF-1α signaling pathway, reducing cellular sensitivity to cuproptosis, thereby protecting bone cells from copper toxicity. This mechanism may represent a self-protective strategy of bone tissue under adverse diabetic conditions and partially explains why diabetic osteoporosis primarily manifests as reduced bone formation rather than enhanced bone resorption.

**Figure 3:**
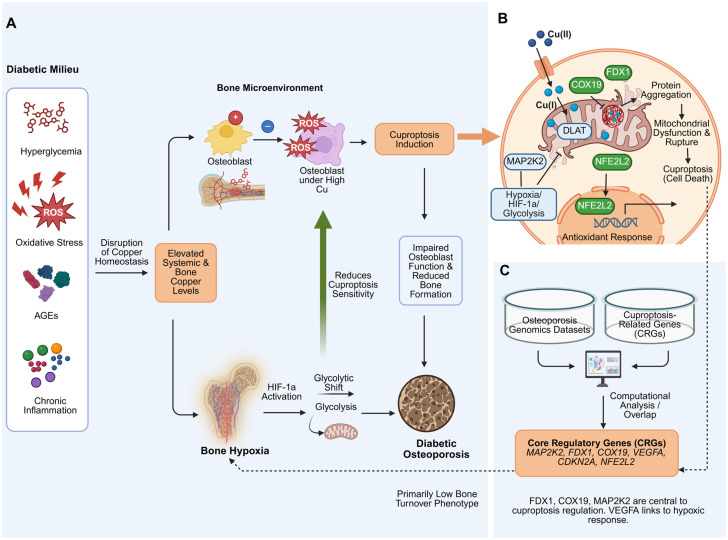
Schematic representation of copper dysregulation and cuproptosis mechanisms in diabetic osteoporosis. (**A**) The diabetic pathological milieu (characterized by hyperglycemia, oxidative stress, AGEs, and chronic inflammation) disrupts systemic copper homeostasis, leading to copper accumulation in the bone microenvironment. Excess copper induces lipid peroxidation and ROS generation, driving osteoblasts towards cuproptosis. Concurrently, the hypoxic bone microenvironment activates HIF-1α-mediated glycolysis, which functions as a compensatory mechanism to attenuate cuproptosis sensitivity. The net result is impaired osteoblast function and low-turnover osteoporosis. (**B**) Detailed molecular mechanism of cuproptosis in osteoblast precursors. Intracellular copper overload targets lipoylated TCA cycle proteins (e.g., DLAT) within mitochondria, leading to protein aggregation and mitochondrial dysfunction. FDX1 and COX19 are identified as key facilitators of this process, while NFE2L2 mediates the antioxidant defense response. The hypoxic glycolytic shift inhibits mitochondrial damage, offering a protective effect. (**C**) Identification of Core Regulatory Genes (CRGs). Intersection analysis of osteoporosis genomic datasets and cuproptosis-related genes reveals six key regulators (MAP2K2, FDX1, COX19, VEGFA, CDKN2A, NFE2L2) that bridge the systemic pathology with the cellular mechanism. Created in BioRender. Chen, W. (2026) https://BioRender.com/wgjly22.

Recent studies have utilized bioinformatics techniques to extract information from existing osteoporosis gene sequencing data and compare it with key regulatory genes (CRGs), identifying six core regulatory genes, including MAP2K2, FDX1, COX19, VEGFA, CDKN2A, and NFE2L2. Reliable evidence indicates that MAP2K2, FDX1, and COX19 are involved in regulating the occurrence of cuproptosis in osteoporosis [10] (Figure 3).

## 4. Copper as a Potential Therapeutic Target for Abnormal Glucose Metabolism and Osteoporosis

### 4.1. Regulation Strategies of Copper Homeostasis

Research on glucose metabolism and bone metabolism has confirmed that copper homeostasis is involved in maintaining glucose metabolism and bone quality. Alterations in copper homeostasis can influence the onset and progression of diseases, highlighting the potential of copper homeostasis as an emerging therapeutic direction. Therefore, researchers have developed various strategies to restore and maintain copper homeostasis.

Currently, two methods are widely used to restore copper balance in the body. The first method involves the use of copper chelators, which are compounds capable of binding to copper ions. They effectively inhibit the accumulation of copper in the body by promoting its excretion. This approach reduces toxicity reactions caused by copper excess while maintaining appropriate copper concentrations in the body [82]. The second method involves the use of copper ionophores, which are specialized molecules that transport copper ions into cells. Copper ionophores can increase intracellular copper levels, thereby ensuring an adequate copper supply within cells and supporting their normal functions. Appropriate copper levels are crucial for maintaining normal glucose metabolism, skeletal health, and promoting bone formation [82,83,84,85].

### 4.2. Copper Ionophores

Copper ionophores can modulate intracellular copper ion concentrations, including members such as elesclomol, dithiocarbamates (DSF), copper(II)(atsm), and copper(II)(gtsm), along with their analogs. These carriers effectively elevate intracellular copper levels through specific mechanisms [86]. Copper ionophores are primarily used in cancer treatment, with relatively extensive research on elesclomol and DSF.

Elesclomol is a copper ionophore specifically targeting mitochondrial metabolism. It induces mitochondrial ROS production and cytotoxic effects [87]. This unique mechanism of action provides a new direction for cancer therapy, particularly for cancer types highly dependent on mitochondrial metabolism [88]. Elesclomol binds to FDX1 in mitochondria and inhibits FDX1-mediated iron–sulfur cluster synthesis, leading to reactive oxygen species production and cancer cell apoptosis [6,89,90]. One study demonstrated that it promotes intracellular copper proliferation, inducing copper-dependent cell death in tumor cells [91]. Furthermore, elesclomol rapidly and selectively transports copper to mitochondria, resulting in mitochondrial copper accumulation, which triggers mitoation therapy of elesclomol, and paclitaxel has shown significant therapeutic effects in patients with metastatic melanoma. Moreover, this combination therapy exhibits similar anticancer effects in various other tumor types [92]. For instance, studies on elesclomol report unique therapeutic advantages in colon cancer, prostate cancer, thyroid cancer, glioblastoma, and ovarian cancer [93,94,95,96,97,98].

DSF, as a copper ionophore, has been extensively studied for its applications in cancer treatment. DSF transports copper into cells, inducing copper-induced apoptosis in cancer cells rich in lipoylated proteins, a finding of great significance for cancer therapy [97,99]. Research indicates that the combination of DSF with copper enhances therapeutic efficacy against certain cancer types, particularly in cancer cells with high ALDH expression, where DSF demonstrates selective targeting and cytotoxic effects [100]. Additionally, DSF can enhance the efficacy of immune checkpoint blockade (ICB) therapy by modulating the tumor immune microenvironment, thereby eliciting systemic immune responses [101]. DSF/Cu combination therapy activates the AKT signaling pathway and reduces the expression level of the tumor suppressor gene PTEN in human breast cancer [102], inhibits the proliferation of diffuse large B-cell lymphoma (DLBCL) cells, and promotes their apoptosis [103]. Notably, the combination of DSF with docetaxel effectively inhibits breast cancer cell growth by suppressing P-glycoprotein (P-gp) and cancer stem cell (CSC) activity [104]. Although DSF offers significant advantages in cancer treatment, it also has certain negative impacts on cell viability. Its cytotoxicity is primarily achieved by increasing ROS levels, inhibiting ALDH activity, and downregulating transcription factor levels, ultimately leading to cell death [105]. Therefore, caution is advised when using DSF.

Although copper ionophores have not been widely applied in the clinical treatment of diabetic osteoporosis, elevated copper levels have been observed in various conditions of glucose metabolism disorders and osteopenia. Compared to other treatment methods, copper ionophores possess unique advantages in increasing intracellular copper content. Thus, developing novel copper ionophores and exploring their applications in diabetic osteoporosis may represent a potential direction for future research.

### 4.3. Copper Chelators

Copper chelators are a class of chemical compounds that form stable complexes with copper ions. Commonly used copper chelators include TETA, triethylenetetramine, tetrathiomolybdate (ATTM), and disodium oxalate. Existing studies have utilized copper chelators to maintain intracellular copper balance, with broad applications in treating disorders such as copper metabolism dysregulation, diabetes, obesity, cancer, neurodegenerative diseases, and cardiovascular diseases [106,107,108].

Copper chelators can halt disease progression by blocking the activity of copper transport proteins. These compounds inhibit lysyl oxidase (LOX) activity and reduce collagen fiber cross-linking, thereby suppressing the process of renal fibrosis [109,110]. The proliferation capacity of vascular endothelial cells is significantly reduced, effectively inhibiting angiogenesis and tumor growth [111]. The copper chelator ATTM markedly inhibits the growth and angiogenesis of hepatocellular carcinoma tumors [112,113]. In diabetic rat models, the copper chelator TETA effectively restores copper balance and myocardial copper content, thereby improving cardiac function [114,115]. Additionally, TETA has potential applications in preventing and treating obesity-related diabetes. It inhibits weight gain (without reducing food intake) by enhancing the activity of spermidine/spermine N1-acetyltransferase 1 (SAT1) and accelerating coenzyme A consumption [106]. Copper in mitochondria can interact with metformin, altering energy metabolism processes by inhibiting complex I of the mitochondrial respiratory chain, revealing a potential link between metformin–copper interactions and diabetes treatment mechanisms [108,116]. Disodium ethylenediaminetetraacetate (EDTA) significantly improves cardiovascular diseases in patients with diabetes and peripheral arterial disease [117]. In bone-related disease research, the early copper chelator D-penicillamine has been clinically used to treat rheumatoid arthritis, showing positive therapeutic effects. A case report indicates that rheumatoid arthritis (RA) patients with heavy metal poisoning who received EDTA treatment showed improvements in symptoms and oxidative stress status [118]. Currently, D-penicillamine is also considered a second-line drug for rheumatoid arthritis [119]. However, the use of copper chelators may lead to excessively low copper levels and cause various side effects. For example, the application of disulfiram in rheumatoid arthritis (RA) treatment often results in hematopoietic system toxicity, accompanied by adverse reactions such as pruritus, various skin rashes, renal impairment, and a metallic taste in the mouth [120].

Therefore, caution is still required when using copper chelators. Nonetheless, copper chelators pose potential risks in DOP. TETA (50 mg/kg/d) in diabetic rats reduces liver copper but simultaneously decreases femoral copper by 15%, leading to a 12% reduction in trabecular bone volume (as detected by μCT) [12], suggesting that systemic chelation may exacerbate bone copper deficiency. Thus, copper chelators are only suitable for DOP subgroups with serum copper > 20 μmol/L and bone copper overload, necessitating the development of bone-targeted chelators.

Copper chelators can bind to excess intracellular copper ions, thereby inhibiting cell proliferation. Furthermore, they can generate reactive oxygen species through redox reactions, inducing apoptosis. However, copper chelators have not been widely applied in models of diabetic osteoporosis, and elevated copper levels have been detected in most patients with abnormal glucose metabolism and reduced bone mass. Therefore, developing novel copper chelators holds great potential and requires further research (Table 2).

## 5. Limitations and Shortcomings

Current research on the association between cuproptosis and DOP remains in the preliminary exploratory stage. For example, Tongying Chen et al. preliminarily revealed the correlation between immune infiltration and cuproptosis-related gene expression in osteoporosis through bioinformatics techniques but did not conduct in vitro functional validation or in vivo experiments, lacking substantial mechanistic research evidence [121]. Haiyang Wu et al., through integrated multi-database analysis, predicted GPR27, PDE8A, NIF3L1, CIR1, and VPS35 as potential therapeutic targets for osteoporosis related to cuproptosis, and qPCR validation in mouse bone marrow mesenchymal stem cells (BMSCs) showed significantly elevated mRNA expression levels of CIR1, NIF3L1, and VPS35, while GPR27 and PDE8A expression decreased [122]. However, this study only provided preliminary evidence at the transcriptional level, lacking protein-level validation and in vivo functional data, and its clinical translational value in human DOP requires further confirmation.

Notably, there is significant controversy regarding the impact of intracellular and extracellular copper levels on diabetes and DOP: on the one hand, some studies suggest that diabetic patients exhibit systemic copper metabolism disorders, where long-term hyperglycemia-induced oxidative stress disrupts the function of copper transport proteins (e.g., SLC31A1, ATP7A), leading to abnormal copper ion accumulation in bone tissue and exacerbating osteoblast damage through cuproptosis; on the other hand, other studies propose that diabetic patients may experience copper deficiency, attributed to impaired copper absorption due to insufficient insulin secretion, and as copper is a component of key enzymes for bone collagen cross-linking, copper deficiency directly affects bone mineralization processes and increases osteoporosis risk. This contradiction may be related to differences in the study populations’ disease stages, glycemic control levels, complication severity, and detection methods. However, it remains unclear whether DOP progression exacerbates copper metabolism abnormalities or whether glucose-lowering or anti-osteoporosis drugs interfere with copper ion homeostasis, necessitating targeted cohort studies for clarification (Table 3).

## 6. Conclusions and Outlook

Diabetic osteoporosis (DOP) represents a core threat to bone health in diabetic patients, with its high fracture risk and bone metabolism disorder characteristics severely impacting patients’ quality of life. This review systematically proposes cuproptosis as a novel form of programmed cell death that may play a key regulatory role in the pathogenesis of DOP, providing a new theoretical perspective and intervention strategy for this field. We emphasize that the pathological progression of DOP not only relies on classical pathways such as the hyperglycemic microenvironment, accumulation of advanced glycation end products (AGEs), oxidative stress, and inflammatory responses, but is also closely linked to the cuproptosis pathway triggered by copper homeostasis imbalance in the diabetic state. Copper ions, by regulating FDX1-mediated abnormal aggregation of lipoylated proteins, degradation of iron–sulfur cluster (Fe-S) proteins, and mitochondrial functional collapse, specifically inhibit osteoblast proliferation and differentiation and disrupt the dynamic balance between bone formation and resorption. This mechanism provides a key molecular explanation for the paradoxical phenomenon of “coexistence of high copper status and suppressed bone formation” in DOP.

Based on the current research status and scientific questions, future studies should focus on the following core directions: clarifying the specific regulatory mechanisms of cuproptosis in DOP, including the interaction network of key molecules such as FDX1 and DLAT in osteoblasts, and elucidating the upstream signals and downstream effector pathways through which copper homeostasis dysregulation triggers cuproptosis; establishing experimental models that accurately simulate the diabetic bone microenvironment; constructing high-glucose-induced in vitro osteoblast or BMSC models and DOP animal models induced by STZ injection in mice to verify the necessity and temporal dependency of cuproptosis in disease onset; utilizing transcriptomic, proteomic, and metabolomic technologies, combined with clinical sample cohorts, to identify specific molecular markers associated with cuproptosis, providing new targets for early screening of DOP; developing novel therapeutic strategies targeting cuproptosis; designing tissue-specific copper chelators (e.g., optimized tetrathiomolybdates) or regulating the SLC31A1/ATP7A copper transport system to remove excess copper ions in bone tissue while preserving their physiological functions; and conducting preclinical studies on cuproptosis inhibitors (e.g., small-molecule compounds targeting FDX1 or the lipoylation pathway) or modulators to construct precision treatment regimens for DOP. In summary, cuproptosis serves as a key bridge connecting copper metabolism disorders and bone metabolism imbalance, holding significant scientific value in the pathogenesis of DOP. Through multidisciplinary integration and in-depth combination of basic and clinical research, targeting the cuproptosis pathway is expected to become a new breakthrough in DOP prevention and treatment, not only deepening the understanding of DOP metabolic regulation mechanisms but also laying a solid foundation for the development of individualized treatment strategies.

## Figures and Tables

**Table 1:** Comparison of Cell Death Mechanisms in DOP Context.

Mechanism	Apoptosis	Ferroptosis	Cuproptosis
Primary trigger in DOP	AGEs, oxidative stress, ER stress	Iron overload, GSH depletion	Copper accumulation
Key executors	Caspases (3/8/9), Bcl-2 family	GPX4 inhibition, ACSL4	FDX1, DLAT/DLST lipoylation
Cellular targets	DNA, mitochondrial outer membrane	Phospholipid membranes	Mitochondrial TCA cycle enzymes
Metabolic state required	Any	Active lipid metabolism	High oxidative phosphorylation
Impact on osteoblasts	Net reduction in cell count (Apoptotic loss)	Membrane integrity loss	Impaired mineralization
Impact on osteoclasts	Variable (depends on stimulus)	May increase activity	Potentially inhibited (at >25 µM Cu^2+^)
Inflammatory response	Minimal (phagocytosed apoptotic bodies)	Moderate (DAMPs release)	Low-to-moderate
Therapeutic targeting	Caspase inhibitors (limited efficacy)	Ferrostatin-1, GPX4 activation	FDX1 inhibitors, copper chelators

**Table 2:** Applications of Copper Chelators in Disease.

Copper Chelators	Type of Model/Subject	Treatment	Conclusion	Refs
DSF ^i^	Animal model of renal injury (UUO + folic acid).	3 mg/kg or 10 mg/kg, once every 3 days.	Inhibits lysyl oxidase to prevent collagen cross-linking; suppresses renal fibrosis.	[111]
TETA ^ii^	Mouse Hepatocellular Carcinoma Xenograft Model.	Oral administration via drinking water (Low: ~195 mg/kg/day; High: ~390 mg/kg/day).	Inhibits angiogenesis and promotes tumor cell apoptosis; suppresses tumor growth in vivo.	[114]
TETA	Diabetes-induced heart failure (Rat model).	Oral administration via drinking water (68 mg/kg/day).	Restores mitochondrial integrity and improves myocardial function; potential target for diabetic heart failure.	[116]
TETA	Obesity-related diabetes.	Daily i.p. injection (100 mg/kg) + 30% sucrose water.	Enhances SAT1 activity and accelerates Acetyl-CoA consumption; inhibits weight gain.	[107]
EDTA ^iii^	Patients with diabetes and PAD (History of MI).	TACT intravenous infusion (up to 3 g edetate disodium).	Reduces relative risk of composite cardiovascular events by 48%.	[118]
EDTA	Rheumatoid arthritis (RA).	2 g/10 mL Calcium Disodium Edetate in 500 mL saline.	Reduces free radical production and improves oxidative stress status.	[119]

^i^. Disulfiram. ^ii^. Triethylenetetramine. ^iii^. Ethylenediaminetetraacetic Acid.

**Table 3:** Current limitations in DOP cuproptosis-related research.

Gap	Current Knowledge	What Is Missing
Bone tissue copper levels	Serum copper elevated in diabetic patients	No data on local copper concentration in trabecular/cortical bone of DOP patients
FDX1 in bone cells	FDX1 is the key cuproptosis regulator in cancer cells	No studies measuring FDX1 expression in osteoblasts from diabetic models
Temporal dynamics	DOP develops over years in diabetic patients	Temporal relationship between copper accumulation and bone loss remains undefined
Cell-type specificity	Copper affects osteoblasts and osteoclasts differently	Unknown which bone cell type is more susceptible to cuproptosis
Signaling crosstalk	AGEs, HIF-1α, and copper metabolism exist in DOP	Interaction between these pathways in triggering cuproptosis unexplored

## Data Availability

Due to its nature as a review article, all references are published articles. The data underlying this article are available in PubMed.

## Abbreviations

ALDH	Aldehyde dehydrogenase
Atox1	Copper chaperone antioxidant 1
ATP7A	Copper-transporting ATPase 1
ATP7B	Copper-transporting ATPase 2
ATTM	Ammonium tetrathiomolybdate
CDKN2A	Cyclin-dependent kinase inhibitor 2 A
CoA	Acetyl-CoA
COX19	Cytochrome C oxidase copper chaperone cox 19
CP	Ceruloplasmin
CRGs	Cuproptosis-relatedgenes
CSCs	Cancer stem cells
CTR1	Copper transporter 1
Cu^2+^	Copper Ion
DLAT	Drolipoamide S-acetyltransferase
DLBCL	Diffuse large B-cell lymphoma
DLST	Dihydrolipoamide S-succinyltransferase
DOP	Diabetic osteoporosis
DSF	Disulfiram
EDTA	Ethylenediaminetetraacetic Acid
FDX1	Ferredoxin 1
Fe-S	Iron–Sulfur Proteins
GDM	Gestational diabetes mellitus
GHRP	Growth hormone-releasing peptide
HbA1c	Glycated hemoglobin
HDL-C	High-density lipoprotein cholesterol
HIF-1	Hypoxia-Induced Factor 1
HSP70	Heat shock protein 70
IGF	Insulin-like growth factor
LIAS	Lipoic acid synthase
LOX	Lysyl oxidase
MAP2K2	Mitogen-activated protein kinase kinase 2
MSCs	Mesenchymal stem cells
MDA	Malondialdehyde
NFE2L2	Nuclear factor, erythroid 2-like 2
P-gp	P-glycoprotein
PTEN	Phosphatase and tensin homolog
RA	Rheumatoid arthritis
ROS	Reactive oxygen species
RUNX2	Runt-related transcription factor 2
SOD	Superoxide dismutase
SAT1	Spermidine/spermine N1-acetyltransferase 1
SLC31A1	Solute carrier family 31 member 1
TETA	Triethylenetetramine
Xc-	System Xc
xCT/SLC7A11	Solute carrier family 7 member 11
